# Rates of injury according to a single or comorbid mental illness identified in a large employee database

**DOI:** 10.1002/1348-9585.12387

**Published:** 2023-01-31

**Authors:** Ray M. Merrill, McKay K. Ashton

**Affiliations:** ^1^ Department of Public Health College of Life Sciences, Brigham Young University Provo Utah USA

**Keywords:** comorbid, employees, health care claims, injury, mental illness

## Abstract

**Objective:**

To identify associations between specific types of mental illness (occurring alone or in combination with other mental illness) and (specific and all types) of injury.

**Methods:**

Analyses involve 21 027 employees aged 18–64 insured by Deseret Mutual Benefit Administrator (DMBA), 2020. Nine classifications of mental illness and 12 classifications of injury are considered. Rate ratios are adjusted for age, sex, and marital status.

**Results:**

The rate of injuries is 13.6%. A positive association exists between any mental illness and injury (rate ratio [RR] = 1.74, 95% CI 1.62–1.87). The positive association is consistent across all types of injury, except burns. While having a mental illness tends to positively associate with having an injury (vs. none), it more strongly associates with having two or more types of injury (vs. none). Injury rates are significantly greater when comorbid mental illness is involved (vs. one type of mental illness), more so for multiple types of injuries. Specifically, there is a positive association between having a mental illness (vs. none) and a single type of injury (vs. none) (RR = 1.58, 95% CI 1.42–1.75) or two or more types of injuries (vs. none) (RR = 1.94, 95% CI 1.70–2.23). Corresponding estimates where comorbid mental illnesses exist (vs. none) are (RR = 2.07, 95% CI 1.70–2.51) and (RR = 3.32, 95% CI 2.64–4.17), respectively. The most common combinations of mental illness that positively associate with injury tend to involve comorbid mental illness.

**Conclusions:**

Several types of mental illness positively associate with injury and are more strongly associated when there is comorbid mental illness.

## 1 INTRODUCTION

Mental illness encompasses a range of mental health conditions that affect mood, thought, and behavior. More common mental illnesses include anxiety, depression, attention deficit hyperactivity disorder (ADHD), bipolar disorder, obsessive–compulsive disorder (OCD), schizophrenia, and autism.^1^ In 2022, almost 20% of adults in the U.S. experienced a mental illness, ranging from 16.4% in New Jersey to 26.9% in Utah.^2^

One study found that those experiencing a mental illness are about 60% more likely to sustain an acute injury than those without a mental illness.^3^ Anxiety, depression, and mania have been associated with an increased risk of falls,^4^, ^5^, ^6^ depressive symptoms have been associated with an increased risk of injury overall,^7^ and ADHD has been associated with an increased risk of injury (particularly fractures and more serious injuries).^8^, ^9^, ^10^, ^11^ Bipolar disorder has been associated with an increased risk of traumatic brain injury (TBI).^12^

Although mental illnesses may be associated with an increased risk of injury, some types of injuries can increase the risk of mental illnesses. The bidirectional relationship between injuries and mental illnesses can be found for several mental illnesses and make causality difficult to identify. TBI may cause anxiety, depression, bipolar disorder, OCD, and schizophrenia.^13^, ^14^, ^15^, ^16^ For example, head injury between ages 11 and 15 years strongly predicted subsequent development of depression, bipolar disorder, and schizophrenia.^16^ On the other hand, a longitudinal study showed that bipolar disorder significantly increased the risk of subsequent TBI.^12^ Depression and depressive episodes have been associated with an increased risk of sustaining an injury, while sustaining an injury has also been associated with an increased risk of developing depression.^7^, ^17^, ^18^ One study concluded that depression and injuries are risk factors for each other, with depression having a slightly higher influence on injury risk compared to injury and depression risk.^19^ Additionally, the development of anxiety and PTSD have also been associated with injuries, while both anxiety and PTSD are associated risk factors for injuries.^4^, ^20^, ^21^

A further issue that complicates the study of mental illness and injury is that injury may be impacted by whether the index mental illness is accompanied by comorbid mental illness. It is not uncommon for individuals to be diagnosed with two or more mental illnesses at the same time. Comorbid mental illness can be influenced by changes in diagnoses after the onset of the index mental illness and also may be due to shared characteristics underlying given types of mental illness.^22^ Cross‐sectional survey data has shown that comorbid mental illness ranges from 45% to 54%,^23^, ^24^, ^25^, ^26^ and that when ADHD is accompanied by comorbid mental illness, there is an even greater risk of injury.^11^

The purpose of this study was to assess associations between several common types of mental illnesses, whether occurring alone or in combination with other mental illnesses, and injury. We hypothesize that mental illnesses are positively associated with specific types of injury and that those with mental illnesses experience a greater number of types of injuries. We further hypothesize that the positive association between mental illness and injury and number of types of injuries is even more pronounced for individuals experiencing comorbid mental illnesses.

## 2 METHODS

### 2.1 Study population

This study is based on full‐time employees receiving health insurance from the Deseret Mutual Benefit Administrator (DMBA). All employees are eligible for comprehensive physical, mental, and dental health insurance; paid sick leave; and many other benefits. The company was established in 1970 to provide health insurance and retirement income to employees and their families of the Church of Jesus Christ of Latter‐day Saints (LDS or Mormons). Electronic claims data were retrieved for 2020. Geographic areas represented by enrollees included Utah (74%), Idaho (9%), Pacific states (9%), and other American states (8%). There were approximately 26% employees, 21% spouses, 49% dependent children, and 4% other (e.g., married child, stepchild, disabled dependent). The current study focuses on full‐time working employees insured through DMBA. These individuals work in the Church education system, seminaries, and institutes (37%); administration and staff; (17%); manual laborers (35%); and in other companies (11%). The distribution of salaries was 23.54% less than $50 000, 41.82% $50 000 to less than $100 000, and 34.65% $100 000 or more. Unfortunately, 2142 (10.19%) of the 21 027 employees had missing salary information. Because of this high number of missing salaries, this variable is not assessed in the current study.

The data did not provide information on race/ethnicity, which may limit generalizability of the results. However, most employees are members of the Mormon Church, wherein the majority of U.S. Church members are white, non‐Hispanic (84%).^27^

The number of employees insured through DMBA dropped for individuals aged 65 and older as they became eligible for Medicare. The database was de‐identified according to Health Insurance Portability and Accountability Act (HIPAA) guidelines.

### 2.2 Data collection

Analyses involved 21 027 employees in 2020, ages 18 through 64. All employees were insured through DMBA. Employee data was matched to automated medical claims records. They were then de‐identified. The research was given exemption status from the Institutional Review Board at the authors' university based on regulatory and institutional criteria.

The International Classification of Diseases, Tenth Revision, Clinical Modification (ICD‐10‐CM) codes were used to classify injuries and selected mental illnesses.^28^ Records were linked using a common identifying number. Codes used to classify injuries were S00‐S09 for head, S10‐S19 for neck, S20‐S29 for thorax, S30‐S39 for abdomen, lower back, lumbar spine, pelvis, and external genitals, S40‐S49 for shoulder and upper arm, S50‐S59 for elbow and forearm, S60‐S69 for wrist, hand, and fingers, S70‐S79 for hip and thigh, S80‐S89 for knee and lower leg, S90‐S99 for ankle and foot, T20‐T28 and T30‐T32 for burns and corrosions, and T36‐T50 for poisoning. Where multiple claims were filed by an individual for the same type of injury, it was only counted once in the numerator of the rate calculation. However, an individual could contribute to more than one type of injury. For example, if a person experienced a head injury and a foot injury in the same year, they were included in the rate calculations for both head injury and foot injury. If any type of injury occurred, then that person was included in the overall injury rate.

Codes used to classify mental illnesses were F0‐F9. Specific types of mental illness presented in this paper are schizophrenia, delusional, and other nonmood‐psychotic disorders (F20‐F29) hereafter called schizophrenia, bipolar disorder (F31), depression (F32‐F33), anxiety (F40‐F41), OCD (F42), autism (F84.0), and ADHD (F90). Injury rates were derived by dividing the number of employees filing one or more claims for an injury over the number of employees. The rate of a mental illness consisted of the number of employees filing one or more claims for a given condition divided by the number of employees. If multiple claims were filed for a given condition, it was only counted once in the numerator of the rate calculation. However, an individual could contribute to more than one type of mental illness. Because those with more serious mental illness are more likely to seek treatment, rates of mental illness and injury may be lower than actually exist. Stigma toward mental illness may further cause lower use of treatment.

Other variables considered in this study were age, sex, and marital status. Classifications for these variables appear in Table 1.

**TABLE 1:** Rate of injury in the Deseret Mutual Benefit Administrators (DMBA) employee population according to selected variables, 2020.

	*N*	Column %	1+ versus 0				
			*N*	Row %	Rate ratio^a^	95% LCL^a^	95% UCL^a^
Mental Illness							
No	17 185	81.73	2065	12.02	1.00	—	—
Yes	3842	18.27	798	20.77	1.74	1.62	1.87
Age (years)							
18–29	2084	9.91	151	7.25	1.00	—	—
30–39	4521	21.5	487	10.77	1.41	1.19	1.69
40–49	5643	26.84	792	14.04	1.85	1.56	2.19
50–64	8779	41.75	1433	16.32	2.13	1.81	2.50
Sex							
Men	14 419	68.57	1979	13.72	1.00	—	—
Women	6608	31.43	884	13.38	1.00	0.93	1.08
Marital status							
Single	4484	21.32	497	11.08	1.00	—	—
Married	16 543	78.68	2366	14.30	1.19	1.08	1.32

^a^ Adjusted for age, sex, marital status, and mental illness.

### 2.3 Statistical techniques

Poisson regression was used to estimate rates. Injury rates per 100 were presented according to age, sex, and marital status. Injury rates were compared across the levels of these variables using rate ratios and corresponding 95% confidence intervals. Rate ratios were adjusted for age, sex, and marital status. Rate ratios compared injuries according to selected mental illnesses, whether the mental illness occurred alone or with other mental illnesses. Percent distributions were used to evaluate the frequency of injury types by mental illnesses. Finally, using stepwise backward selection, each mental illness was regressed on the 12 types of injury in order to identify those injuries significantly associated with each mental illness. Two‐sided tests of significance were used based on the 0.05 level. Statistical analyses were derived from Statistical Analysis System (SAS) software, version 9.1 (SAS Institute Inc.).

## 3 RESULTS

**FIGURE 1:**
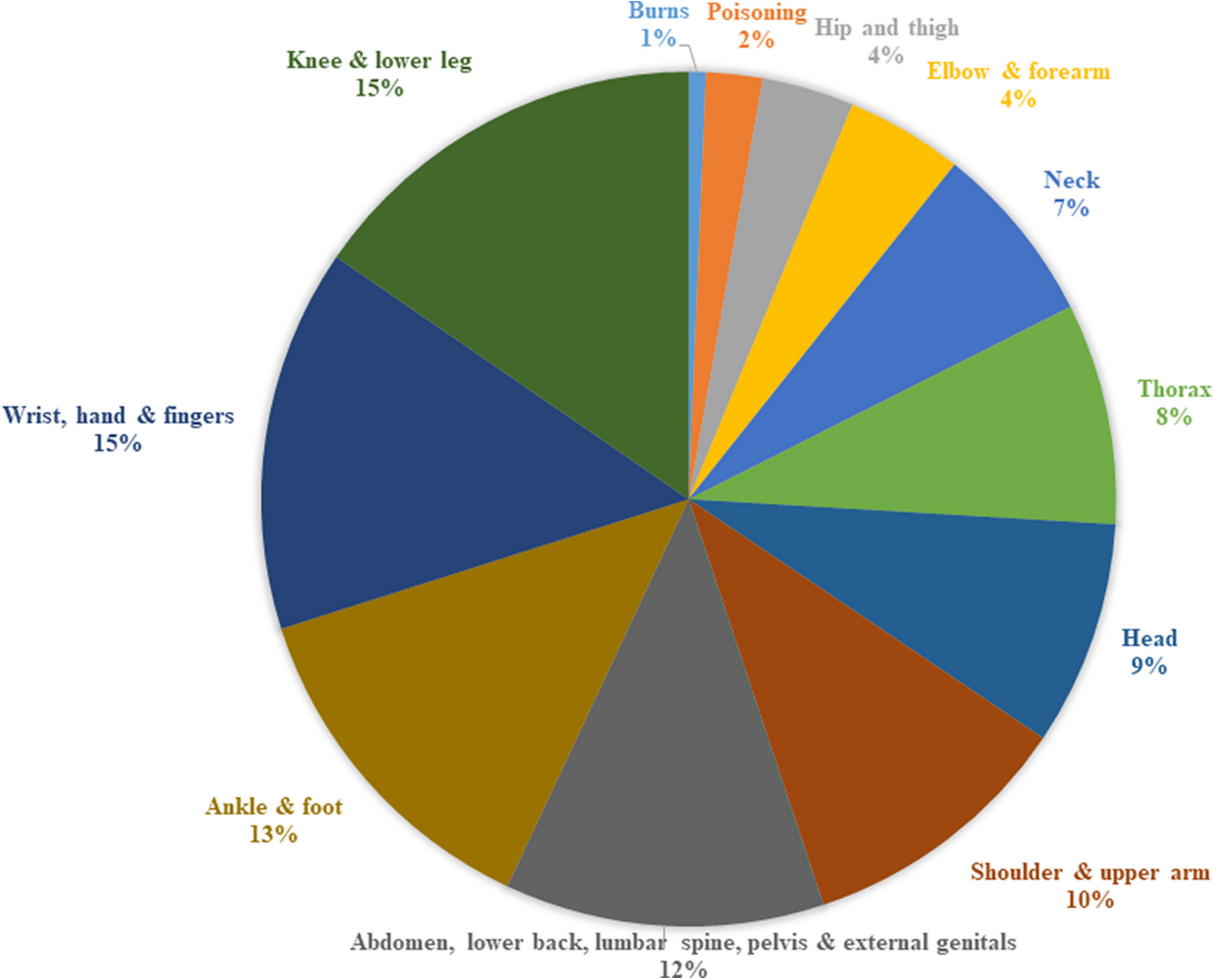
Types of injury claims among employees reported in the Deseret Mutual Benefit Administrators (DMBA) population, 2020.

The number of employees in 2020 who filed a claim for one or more types of injuries is 2863 (13.6%). The distribution of types of injuries appears in Figure 1. The most common classification of injury involved the knee and lower leg, followed by the wrist, hand, and fingers.

Injury rates by age, sex, marital status, and mental illness are shown in Table 1. There is a significant positive association between filing a claim for one or more types of mental illness (vs. none) and having one or more types of injuries (vs. none). The rate of one or more types of injury increase with age and in married (vs. singles). Sex is not significantly associated with having one or more types of injury.

Variables significantly associated with specific types of injuries are shown in Table 2. Mental illness and age are consistently positively associated with the various types of injury. Women are less likely than are men to experience injuries of the abdomen and lower back and wrist, hand, and fingers. On the other hand, they are more likely to experience ankle and foot injuries. Married are more likely than singles to experience injuries of the wrist, hand, and fingers and hip and thigh.

**TABLE 2:** Variables associated with specific types of injuries.

Type of injury	Head	Neck	Thorax	Abdomen, lower back …	Shoulder and upper arm	Elbow and forearm	Wrist, hand and fingers	Hip and thigh	Knee and lower leg	Ankle and foot	Burns	Poisoning
Mental Illness	✓+	✓+	✓+	✓+	✓+	✓+	✓+	✓+	✓+	✓+		✓+
Age	✓+	✓+	✓+	✓+	✓+	✓+	✓+	✓+	✓+	✓+	✓+	✓+
Women versus Men				✓‐			✓‐			✓+		
Married versus Singles							✓+	✓+				

*Note*: ✓ indicates significance at the 0.05 level, based on the chi‐squared test. “+” refers to a positive association and “–” refers to a negative association. Each injury type was simultaneously regressed on age, sex, marital status, and mental illness.

Injury rates for selected mental illnesses are shown in Table 3. Associations tend to be positive between the mental illnesses and one type of injury (vs. none) and two or more types of injuries (vs. none). The latter associations are significantly stronger for anxiety, depression, stress, OCD, and autism.

**TABLE 3:** Rate of injury according to mental illness, 2020.

Mental illness	*N*	%	1 versus 0			2+ versus 0			2+ versus 1		
			Rate ratio^a^	95% LCL^a^	95% UCL^a^	Rate ratio^a^	95% LCL^a^	95% UCL^a^	Rate Ratio^a^	95% LCL^a^	95% UCL^a^
Anxiety	1995	9.49	1.63	1.46	1.83	2.46	2.03	2.97	1.34	1.13	1.60
Depression	1791	8.52	1.62	1.44	1.82	2.25	1.84	2.77	1.26	1.05	1.51
Stress	478	2.27	1.41	1.13	1.78	2.96	2.18	4.03	1.68	1.29	2.18
ADHD	422	2.01	1.69	1.37	2.09	1.39	0.84	2.29	0.80	0.50	1.28
Bipolar disorder	123	0.58	1.95	1.37	2.78	3.68	2.15	6.30	1.49	0.93	2.39
OCD	84	0.40	1.38	0.77	2.45	3.85	2.01	7.37	2.12	1.26	3.56
Schizophrenia	23	0.11	1.63	0.67	3.96	—	—	—	—	—	—
Autism	7	0.03	1.84	0.32	10.50	9.40	3.08	28.64	3.18	1.39	7.27
Other	340	1.62	1.61	1.27	2.04	1.99	1.28	3.09	1.11	0.74	1.64

^a^ Adjusted for age, sex, and marital status.—insufficient numbers to compute.

Injury rates for a single type or multiple types of mental illnesses compared (vs. none) are shown in Table 4. Associations between having a mental illness (vs. none) and having a single type of injury (vs. none) or multiple types of injury (vs. none) are significantly positive, significantly more so in the latter case. Rate ratios of injury are significantly greater when two or more types of mental illness are involved (vs. one type of mental illness). Interaction terms between the mental illness variable and age, sex, and marital status are not statistically significant.

**TABLE 4:** Rate of injury according to a single or multiple types of mental illnesses (vs. none), 2020.

Mental Illness	*N*	%	1 versus 0			2+ versus 0			2+ versus 1		
			Rate ratio^a^	95% LCL^a^	95% UCL^a^	Rate ratio^a^	95% LCL^a^	95% UCL^a^	Rate ratio^a^	95% LCL^a^	95% UCL^a^
None	17 185	81.73	1.00	—	—	1.00	—	—	1.00	—	—
1	2685	12.77	1.58	1.42	1.75	2.07	1.70	2.51	1.21	1.02	1.44
2+	1157	5.50	1.94	1.70	2.23	3.32	2.64	4.17	1.46	1.19	1.79
Chi‐squared *P*‐value			.0214			.0007			.2238		

^a^ Adjusted for age, sex, and marital status.

Twenty‐one of the most common combinations of types of mental illness are positively associated with having one or more types of injuries (vs. none) in Table 5. Small numbers limit identifying statistical significance for some of these combinations. Those with claims filed for both anxiety and bipolar disorder have the strongest association with injury (vs. no injury). Nine of the largest 10 rate ratios involve multiple mental illnesses. Fourteen of the largest 21 rate ratios involve combinations of mental illnesses.

**TABLE 5:** Rate of one or more injuries (vs. none) according to mental illness, 2020.

Mental illness	*N*	%	1+ versus 0		
			Rate ratio^a^	95% LCL^a^	95% UCL^a^
None	17 185	81.73	1.00	—	—
Anxiety and bipolar	27	0.13	3.18	2.02	5.00
Anxiety, depression, stress, ADHD	8	0.04	2.76	1.13	6.75
Anxiety, depression, and stress	78	0.37	2.70	1.91	3.82
Anxiety and OCD	13	0.06	2.65	1.20	5.83
Depression and OCD	8	0.04	2.63	0.77	8.92
Depression and stress	50	0.24	2.45	1.58	3.80
Depression and ADHD	62	0.29	2.36	1.58	3.54
Anxiety, depression, and bipolar	19	0.09	2.21	1.03	4.71
Anxiety and depression	608	2.89	2.00	1.72	2.33
ADHD	208	0.99	1.99	1.54	2.57
Depression and bipolar	12	0.06	1.96	0.76	5.08
Bipolar	31	0.15	1.96	1.03	3.73
Other	340	1.62	1.82	1.49	2.23
Anxiety and ADHD	59	0.28	1.68	0.98	2.86
Anxiety	999	4.75	1.67	1.46	1.90
Anxiety and stress	67	0.32	1.65	0.99	2.75
Anxiety, depression, and ADHD	50	0.24	1.62	0.89	2.93
Stress	235	1.12	1.57	1.20	2.06
Depression	839	3.99	1.52	1.31	1.76
OCD	28	0.13	1.39	0.57	3.40
Anxiety, depression, and OCD	18	0.09	1.25	0.36	4.40

^a^ Adjusted for age, sex, and marital status.

## 4 DISCUSSION

It is well known that mental illnesses are associated with injuries and that the causal direction is bidirectional.^3^, ^4^, ^5^, ^6^, ^7^, ^8^, ^9^, ^10^, ^11^, ^12^, ^13^, ^14^, ^15^, ^16^, ^17^, ^18^, ^19^, ^20^, ^21^ It is also well known that specific mental illnesses are often accompanied by comorbid mental illness and that more severe mental illnesses have a greater likelihood of comorbid mental illness.^22^, ^23^, ^24^, ^25^, ^26^ What is less well known is whether mental illness is associated with specific types of injury and the number of types of injuries. It is also not clear whether the addition of comorbid mental illness to the index mental illness further increases the rate of injury or the number of types of injuries.

The current study hypothesizes that mental illness is positively associated with injury and that those with mental illness experience a greater number of types of injuries. The study further hypothesizes that the positive association between mental illness and injury and number of types of injuries is even greater for individuals with comorbid mental illnesses. The results of this study support these hypotheses.

Our data represents information about mental illness and injury for which treatment was received. Mild cases may not have pursued treatment such that our rates may be lower than reflected in cross‐sectional surveys. It is also possible that individuals with mental illnesses delay or do not seek treatment or prematurely end treatment for fear of discrimination and labeling, or believe the treatments are not effective.^29^ Nevertheless, the rate of mental health treatment among adults 18–64 in the U.S. in 2020 was 20.5%,^30^ only slightly higher than the rate of mental health treatment of 18.3% found in this study.

Mental illness consistently positively correlated with all of the specific injury types considered, except burns, and age consistently positively correlated with all the injury types. Small numbers may explain why the correlation involving burns did not reach statistical significance. Increasing injury rates in middle‐aged individuals is consistent with a study showing the frequency of falls rising from young age to middle age to old age.^31^ The observed positive association between age and poisoning is consistent with adults in their 50 s and older being more vulnerable to serious drug interactions and side effects. This is because of reduced muscle mass and increased fat, as well as reduced kidney and liver function. Further, repetition of intentional self‐poisoning could be associated with underlying mental health concerns.^32^ Those with mental illnesses are more likely to misuse drugs,^33^ which could account for a large portion of the poisoning injuries.

This study found no significant association between sex and injury overall, but that men had higher rates of injury than women of the abdomen, lower back, spine, and pelvis; and wrist, hands, and fingers. These higher rates in men are consistent with other research showing that they have higher rates of injury and greater severity of injury.^34^ However, our results showed that men were less likely to experience ankle and foot injuries.

Higher injury rates in married employees is inconsistent with a study conducted in New Zealand in which never married (vs. married) had twice the risk of injury from motor vehicle accidents.^35^ Yet, in the current study married employees had a greater risk of less serious types of injuries, where a married spouse may have provided encouragement to their partner to seek care.

Each mental illness was associated with significantly higher injury rates. Other research has similarly identified associations between these types of mental illness and injuries.^4^, ^5^, ^6^, ^7^, ^8^, ^9^, ^10^, ^11^, ^12^, ^13^, ^14^, ^15^, ^16^ Each association likely involves a bidirectional relationship. For example, anxiety may cause increased muscle tension and coordination difficulties, which increase the risk of injury.^36^ On the other hand, injury can increase the risk of stress, anxiety, and depression.^37^ In addition, the results found that mental illness makes people more prone to multiple types of injuries compared to just one type of injury. This result complements other research showing that mental illness is associated with more serious injuries and longer injury‐related hospital stays.^9^, ^10^, ^11^, ^12^, ^38^

Injury rates for each mental illness are greater when comorbid mental illnesses exist. This implies that a more serious mental illness status (i.e., more than one type) is associated with a higher rate of injury. It is possible that multiple, interacting medications contribute to this result. Anxiolytic, antidepressant, and antipsychotic medications have been found to significantly increase the risk of TBI in those with a mental illness.^39^ In a study of individuals 0–18, the use of ADHD and concomitant psychotropic (versus only ADHD drugs) significantly increased the risk of injuries.^40^ This increased risk of injury could be due to interacting medications, or it may be that those with more severe forms of their respective mental illnesses are already more likely to sustain an injury and are medicated due to their severity.

Approximately 14% of all injuries involved the head. Head injury was statistically associated with each of the mental illnesses studied, except for OCD. The associations with head injury are explained by head injuries both causing mental illness but also being the result of it.^7^, ^12^, ^14^, ^15^, ^16^

## 5 LIMITATIONS AND STRENGTHS

This study is based on employee medical claims in the DMBA database during 2020. As all employees are enrolled in DMBA, selection bias of subgroups is not a problem. A mental illness diagnosis will not lead to losing insurance, so making selective underreporting of mental illness claims, for this reason, is unlikely. However, it may be that some individuals experiencing mental illnesses did not file a claim because of fear of discrimination and labeling or because treatments were not thought to be effective. Incorrect diagnosis and treatment of mental illness is also possible. The available data did not allow us to identify the level of underreporting of mental illnesses or injuries. The fact that less serious mental illnesses may not be captured in the claims database is less likely a problem regarding the measures of association, if the level of underreporting is similar between mental illness and injuries. Further, we do not know whether the injuries occurred on the job or elsewhere. Most of the white‐collar jobs likely occurred outside of the workplace. Finally, the database did not allow us to evaluate the temporal relationship between mental illnesses and injury. Hence, drawing conclusions about causal direction are not possible.

## 6 CONCLUSION

Each mental illness is individually associated with an increased rate of all major types of injury. The mental illnesses are more strongly associated with the occurrence of two or more types of injuries. There is a greater association with the rate of injury when comorbid mental illnesses are present. Comorbid mental illnesses are more strongly associated with the presence of two or more types of injuries. This implies that a more serious impaired mental illness state contributes more to a greater rate of injury and more types of injury being involved.

## AUTHOR CONTRIBUTIONS

Both authors have contributed to the conception or design of the work, drafted the work or revised it critically for important intellectual contents, approved the version to be published, and agreed to be accountable for all aspects of the work. RMM analyzed the data.

## CONFLICT OF INTEREST STATEMENT

No conflict of interest declared.

## Data Availability

The datasets generated and analyzed during the current study are not publicly available due to confidentiality restrictions but are available in a de‐identified and aggregated format from the corresponding author on reasonable request. The data were made available to the authors by DMBA strictly for research purposes.
